# Polydrug use with opioid involvement: Results from a national sample of U.S. civilians aged 12 years or older

**DOI:** 10.1371/journal.pone.0345058

**Published:** 2026-03-18

**Authors:** Kele Ding, Saroj Bista, Trisha Welter, Krista K. Wheeler, Gary A. Smith, Jingzhen Yang

**Affiliations:** 1 Health Education and Promotion Program, School of Health Sciences, Kent State University, Kent, Ohio, United States of America; 2 Center for Injury Research and Policy, Abigail Wexner Research Institute, Nationwide Children’s Hospital, Columbus, Ohio, United States of America; 3 Student Wellness, University of Iowa, Iowa City, Iowa, United States of America; 4 Department of Pediatrics, College of Medicine, The Ohio State University, Columbus, Ohio, United States of America; Chuo University, JAPAN

## Abstract

This study examined the prevalence and patterns of polydrug use among a national sample of civilians aged 12 years or older, with a focus on polydrug use involving opioids, assessing the influence of the first drug used on these behaviors. Using 2020 National Survey on Drug Use and Health (NSDUH) data, five mutually exclusive drug groups were identified in a hierarchical order: opioid use, stimulant or psychoactive drug use, non-opioid prescription drug misuse, marijuana use, and legal substance use. Polydrug use was defined as the use of drugs from two or more groups. Among 32,893 participants, the prevalence of past-month polydrug use (but not within the past year) was 11.4%, while past-year use (but not past month) was 2.8%. Among participants who used opioids in the past month, 89.2% engaged in polydrug use; among those with past-year opioid use, 48.0% engaged in polydrug use. Of those engaging in past-month and past-year polydrug use, 7.0% and 32.5% used opioids, respectively. Participants who identified as female and Hispanic had lower odds of polydrug use than their respective counterparts. Respondents who initiated drug use with opioids had over five times higher odds of past-month (odds ratio [OR] = 5.45, 95% confidence interval (CI): 2.61–11.37) and past-year **(**OR = 5.58, 95% CI: 2.76–11.29) polydrug use than those whose first use involved legal substance use. Their odds of opioid-involved polydrug use were 15.56 times higher for past-month use **(**95% CI: 4.92–49.18) and 54.66 times higher for past-year use **(**95% CI: 23.80–125.55). While opioid use in the general population is low, it is highly prevalent among those engaging in polydrug use with opioids as their first drug. Efforts to prevent polydrug use should prioritize those who identify as male or whose first drug was opioids, using targeted education, harm reduction, and other prevention strategies.

## Introduction

Polydrug use, defined as the use of two or more substances together within a short time [1,2], is linked to high mortality rates [3]. The combined effects of multiple drugs can exceed those of a single substance use [1]. As a complex phenomenon, polydrug use patterns in individuals varies by factors such as price, availability, legal status, method of administration (e.g., non-injection vs. injection), and the contexts in which the drugs are used [4,5]. Over the years, researchers have used various terms to describe specific polydrug use patterns, including concurrent use (i.e., use of multiple drugs within overlapping time periods, regardless of whether they were taken in the same sitting) and simultaneous use (i.e., use of multiple drugs in the same sitting or occasion) [4]. However, there is no standardized approach for categorizing and studying multiple substance use behaviors. Existing studies have often focused on the co-use of alcohol and other drugs [6,7], including co-use of alcohol with tobacco, cannabis, cocaine, or prescription stimulants [8–10], or on specific populations such as youth, adults, or people who use opioids [7,11–13]. There is a lack of studies examining specific combinations or patterns of multiple drug use and the associated risk factors in the general population [14].

To address this gap, it is important to examine an individual’s substance use recency, as the timing of use provides valuable insight into behavioral patterns and risk trajectories [14,15]. Past-month use typically reflects more recent and potentially ongoing substance use, whereas past-year use (but not past month) may represent intermittent or experimental patterns [16]. Distinguishing between these two time frames helps clarify the intensity and persistence of a person’s substance use behaviors and how early use may predict continued or escalating risk [17,18]. Understanding patterns of both past-month and past-year polydrug use can also help identify emerging trends, informing public health priorities, and guiding targeted prevention strategies [19].

A growing body of evidence indicates that polydrug use is rising nationally, largely in the context of the ongoing opioid epidemic [20–22]. Polydrug use is highly prevalent among individuals who use opioids, with opioids commonly co-used with stimulants, alcohol, benzodiazepines, and other psychoactive substances. More than half of the opioid overdose deaths in the United States involve cocaine, methamphetamine, or benzodiazepines [23–25] and nearly half of all drug overdose deaths involved multiple substances [26–28], underscoring the central role of opioids in polysubstance-related mortality. The literature consistently shows that polydrug use is the norm rather than the exception among people who use opioids. Opioids are frequently co-used with other substances to enhance euphoria, manage withdrawal symptoms, or modulate drug effects [11,13,29]. These patterns suggest that opioid use is embedded within broader substance use trajectories rather than occurring in isolation.

Focusing on individuals whose first substance use involved opioids is particularly important, as early opioid initiation may signal heightened vulnerability to subsequent polydrug use and more severe risk profiles, including overdose and adverse mental, behavioral, and social outcomes [2,30–32]. Understanding how opioid initiation relates to later polydrug use can help identify high-risk pathways and inform more targeted prevention and intervention strategies.

Methodological challenges, including the lack of standardized definitions and measures and consensus on co-occurring substance use, contribute to significant variations across studies [33]. Additional obstacles in polydrug use research include small subsample sizes that hinder complex statistical analyses [34] and skewed distributions that complicate hierarchical multilevel regressions, limiting interpretability [35]. These challenges highlight the need for improved methodological approaches, including the use of large, nationally representative samples to more accurately assess polydrug use patterns, opioid involvement, and timing of use, patterns that are difficult to examine in smaller or clinical samples.

This study aimed to examine the prevalence and patterns of polydrug use, with a focus on respondents with polydrug use involving opioids, among a nationally representative sample of the civilian, non-institutionalized population aged 12 years or older. Additionally, it assessed the associations between the type of drug first used and subsequent polydrug use patterns. Specifically, using National Survey on Drug Use and Health (NSDUH) data, this study addressed the following research questions: What was the prevalence of polydrug use? What were the behavioral patterns of polydrug use, especially when involving opioid use? What were the odds of opioid involvement in polydrug use? What association was observed between initial drug type and later polydrug use?

## Materials and methods

### Data source

This study analyzed cross-sectional data from the 2020 NSDUH, an annual survey sponsored by the Substance Abuse and Mental Health Services Administration (SAMHSA). The NSDUH includes measures on the prevalence and correlates of drug use in a nationwide community sample, with data collection methods detailed elsewhere [36]. Briefly, the NSDUH conducts annual surveys among the civilian, non-institutionalized population aged 12 years or older in the United States. Prospective participants receive a lead letter with introductory information and are then approached by a door-to-door field interviewer to complete the survey. As the leading data source on drug use in the United States, the NSDUH has been repeatedly used for studies of polydrug use [37].

The 2020 NSDUH was the most recent publicly available, deidentified data at the time of this study, including a national sample of 32,893 survey respondents. For this study, we analyzed the 2020 NSDUH respondents’ use of the following 12 drug types: alcohol, marijuana, cocaine (including crack), methamphetamine, hallucinogens, heroin, inhalants, tobacco, pain relievers, tranquilizers, stimulants, and sedatives. This study was reviewed by the authors’ institutional review board and determined as not research involving human subjects (IRB ID: STUDY00003531).

### Outcome variables and measures

#### Drug use.

In the NSDUH, respondents’ most recent use or misuse of a drug was classified into three mutually exclusive categories [19]:

1. ever use or lifetime use, defined as last use or misuse occurring more than 12 months ago (i.e., not within the past year);
2. past-year use, defined as last use or misuse occurring more than 30 days ago but within the past 12 months; and
3. past-month use, defined as last use or misuse occurring within the past 30 days.

For the present study, drug use was operationalized into two mutually exclusive binary variables: past-month use (Yes vs. No) and past-year use (Yes vs. No).

#### Polydrug use.

**Fig 1:**
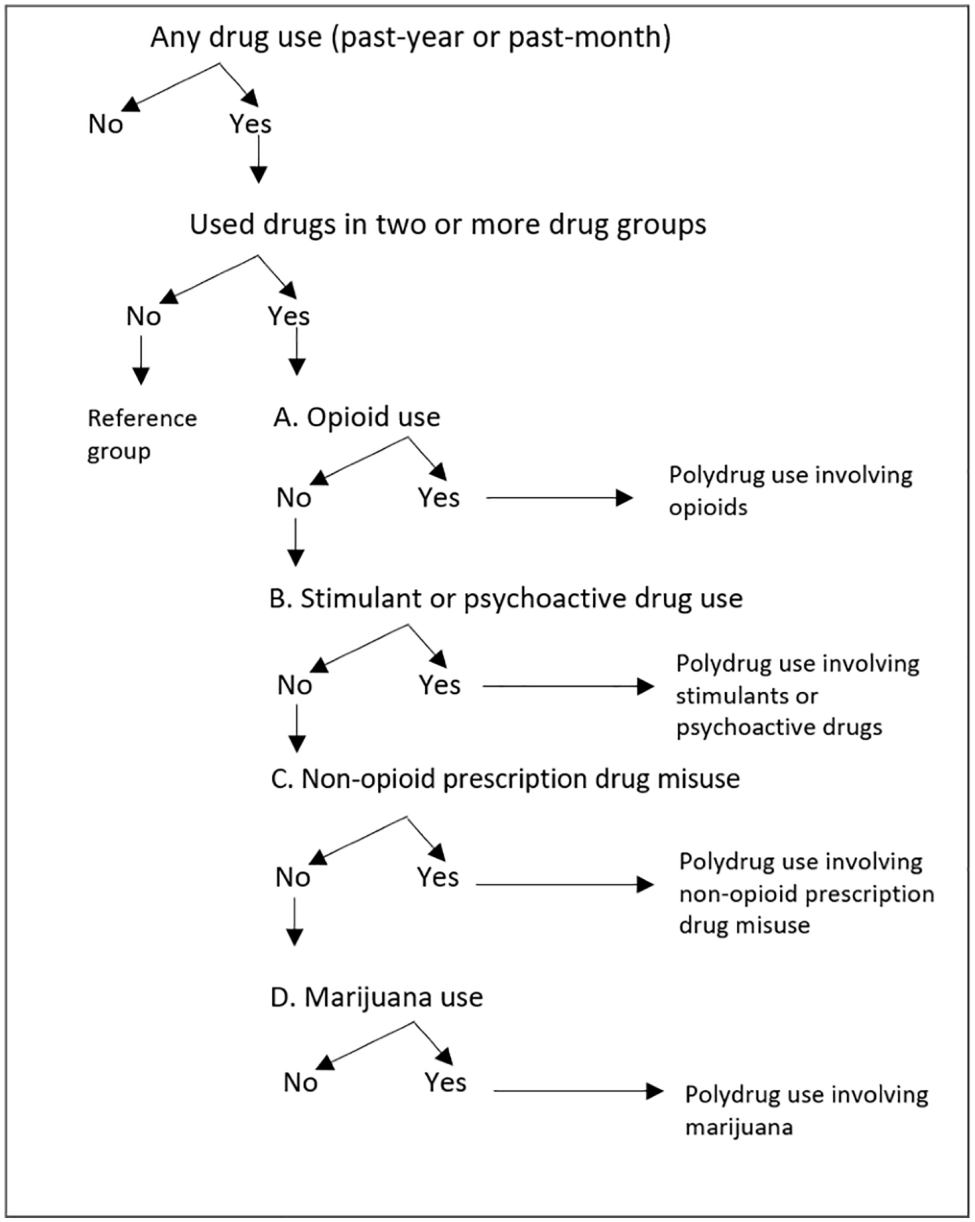
Hierarchical decision tree that classifies polydrug use.

The NSDUH includes 12 drug types (see description above). To examine the prevalence of polydrug use, we classified these 12 drug types into five groups based on their popularity and legal status, excluding prescription drug used as directed for medical purposes [33,38]. This classification emphasized drug-use behaviors, with particular attention to the illegal use or misuse of prescription opioid pain relievers. For the purposes of this study, “legal substances” were defined based on the regulatory status of the products themselves, rather than the legality of a specific use event. This term referred to substances that were legally manufactured, sold, or prescribed under U.S. federal law (e.g., alcohol, nicotine, inhalants). Unlike illicit controlled substances such as cocaine or heroin, these substances were commercially available and legal to possess. The five drug groups were coded hierarchically and were mutually exclusively (see Fig 1).

1) Opioid use: Use of heroin and misuse of prescription opioid pain relievers.
2) Stimulant or psychoactive drug use: Use of cocaine (including crack), methamphetamine, and/or hallucinogens (including LSD, PCP).
3) Non-opioid prescription drugs misuse: Misuse of non-opioid pain relievers, stimulants, sedatives, and/or tranquilizers.
4) Marijuana use. Use of marijuana.
5) Legal substances use: Use of alcohol, tobacco, and/or inhalants.

We classified each of included study participants into one of the three groups: 1) *Polydrug use*: Individuals who used or misused drugs in two or more of the above five drug groups, 2) *Single drug use*: Individuals who used or misused only one of the five drug groups, and 3) *No drug use*: Individuals who did not use or misuse any drugs in the five drug groups. We analyzed polydrug use as use in the past year or in the past month.

#### Opioid use.

In the NSDUH dataset, each of the 12 prescription opioid pain relievers (i.e., hydrocodone, oxycodone, Percocet, tramadol, codeine, morphine, fentanyl, buprenorphine, oxymorphone, hydromorphone, methadone, and Demerol) was specifically marked as either used or misused by respondents in the past year. Thus, any of these 12 prescription opioid pain relievers marked as misused (i.e., used in a way not recommended by a doctor) were included as past-year misuse of opioids. We combined heroin use and the misuse of prescription opioid pain relievers under the category of opioid use. We defined *past-year opioid use* as a “yes” response to the questions about “use of heroin in the past year” or “misuse of any prescription opioid pain relievers in the past year.”

The 2020 NSDUH only asked respondents about any pain reliever use and misuse in the past month without specifying whether it was an opioid pain reliver or not. Therefore, we combined two questions to define any prescription opioid pain reliever misuse in the past month. We defined *past-month opioid use* as a “yes” response to the questions about “use of heroin in the past month” or “misuse of any pain reliever in the past month” among respondents who responded “misused” any prescription opioid pain relievers in the past year.

#### Patterns of polydrug use.

**Fig 2:**
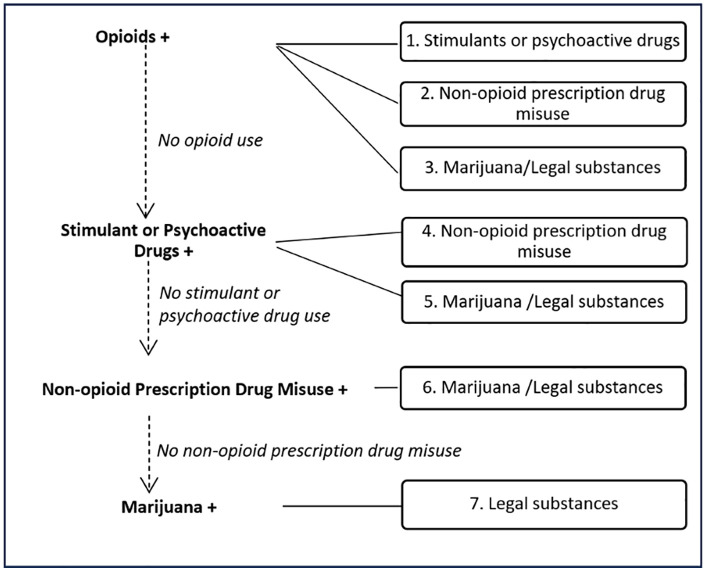
Seven distinct polydrug use patterns.

We utilized a hierarchical decision tree to classify the polydrug use of individuals based on five drug groups (Fig 1). This approach identified seven distinct polydrug use patterns, each defined by unique, mutually exclusive combinations of these groups for analysis. (Fig 2). The seven distinct polydrug use patterns are as follows:

Polydrug Use involving Opioids:

1. *Opioids and Stimulants or psychoactive drugs:* Individuals who used defined opioids and stimulants or psychoactive drugs concurrently.
2. *Opioids and Non-opioid prescription drug misuse:* Individuals who engaged in defined opioid use and non-opioid prescription drug misuse concurrently.
3. *Opioids and Marijuana/Legal substances:* Individuals who used defined opioids, and marijuana and/or legal substances concurrently.

Polydrug Use involving Stimulants or Psychoactive drugs (other than opioids):

4. *Stimulants or psychoactive drugs and Non-opioid prescription drug misuse:* Individuals who engaged in stimulants or psychoactive drugs and non-opioid prescription drug misuse concurrently.
5. *Stimulants or psychoactive drugs and Marijuana/Legal substances:* Individuals who used a stimulants or psychoactive drugs, and marijuana and/or legal substances concurrently.
6. *Polydrug Use involving Non-opioid prescription drug misuse and Marijuana/Legal substances:* Individuals who engaged in non-opioid prescription drug misuse and marijuana use and/or legal substance use concurrently.
7. *Polydrug Use involving Marijuana and Legal substances:* Individuals who used marijuana and legal substances concurrently.

### Other variables

#### Demographic variables.

Demographic variables included in the analysis were: age (five categories: 12–17; 18–25; 26–34; 35–49; 50 or older), sex (male, female), and racial/ethnic identity. The original variable of racial and ethnicity had eight categories, and we collapsed some of the categories based on the distributions to form five categories: 1) White Non-Hispanic, 2) Black Non-Hispanic, 3) Other Race Non-Hispanic, 4) Multi-race Non-Hispanic, and 5) Hispanic.

#### First drug used.

We also included a variable, first drug used, in the analysis, which was defined based on the self-reported age of first-time use of a drug among the five drug groups.

### Data analysis

We first classified each survey respondent by the five-group drug use status in the past-month (but not within the past year) or past-year (but not past month). We then classified respondents who used at least one of the five drug groups by polydrug use categories and patterns. Descriptive analyses were conducted to describe the distributions of demographics for the survey respondents, as well as the prevalence of polydrug use, including whether it was polydrug use or single drug use, and whether use occurred in the past month or in the past year, using both unweighted and weighted data. Further, we described the patterns of polydrug use, including whether polydrug use involved opioids.

Logistic regression was used to examine the odds of polydrug use vs. single drug use among respondents who used at least one of the five drug groups, as well as the odds of polydrug use with opioid involvement vs. without opioid involvement among respondents who used two or more of the five drug groups. Additionally, we examined associations between different types of first drug used and the odds of polydrug use or polydrug use involving opioids. We conducted separate logistic regressions for respondents who used drugs in the past month and in the past year, respectively. Model tests were carried out in two rounds. The first round tested the null model without covariates. The second round adjusted for demographic variables including sex, age, and race/ethnicity. All analyses were conducted in SAS and completed by August 2024. The significance level was set at 0.05.

## Results

### Demographic characteristics of respondents

Of the 32,893 NSDUH 2020 respondents, 54% identified as male and 46% as female (Table 1). The age distribution was 17.4% in both the 12–17 and 26–34 age groups and 24.2% in the 18–25 group. Nearly two-thirds of respondents (64.9%) identified as non-Hispanic Whites, followed by identification as Hispanic (14.9%) and as non-Hispanic Blacks (9.2%).

**Table 1:** Demographics and prevalence of drug use among the respondents, 2020 NSDUH.

	Unweighted, n (%)	Weighted, n (%)
**Total** ^**1**^	32,893	276,911,975
**Sex**		
Male	15,130 (46.0)	134,390,922 (48.5)
Female	17,763 (54.0)	142,521,053 (51.5)
**Age Category**		
12-17	5,723 (17.4)	24,982,702 (9.0)
18-25	7,946 (24.2)	33,497,019 (12.1)
26-34	5,711 (17.4)	40,525,872 (14.6)
35-49	7,418 (22.6)	61,886,651 (22.3)
50 or older	6,095 (18.5)	116,019,731 (41.9)
**Race/ Ethnicity**		
White Non-Hispanic	21,362 (64.9)	170,743,481 (61.7)
Black Non-Hispanic	3,025 (9.2)	33,619,046 (12.1)
Other Race Non-Hispanic^2^	2,281 (6.9)	19,028,019 (6.9)
Multi-race Non-Hispanic	1,329 (4.0)	5,141,493 (1.9)
Hispanic	4,896 (14.9)	48,379,936 (17.5)
**Past-Month Drug Use**		
No drug use	14,348 (43.6)	116,532,264 (42.1)
Single drug use	14,371 (43.7)	128,932,228 (46.6)
Polydrug use^3^	4,174 (12.7)	314,47,483 (11.4)
**Past-Month Use of Five Drug Groups** ^**4**^		
No drug use	14,348 (43.6)	116,532,264 (42.1)
Opioid use (heroin and misuse of opioid prescription pain relievers)	288 (0.9)	2,397,454 (0.9)
Stimulant or psychoactive drug use (non-opioid)	413 (1.3)	4,092,273 (1.5)
Non-opioid prescription drug misuse	573 (1.7)	3,578,622 (1.3)
Marijuana use	4,421 (13.4)	31,966,816 (11.5)
Legal substance use (alcohol, tobacco, and/or inhalants)	17,848 (54.3)	155,727,429 (56.2)
**Past-Year Drug Use (but not past month)**		
No drug use	24,906 (75.7)	217,633,962 (78.6)
Single drug use	6,734 (20.5)	51,561,632 (18.6)
Polydrug use^3^	1,253 (3.8)	7,716,381 (2.8)
**Past-Year Use (but not past month) of Five Drug Groups** ^**4**^		
No drug use	24,906 (75.7)	217,633,962 (78.6)
Opioid use (heroin and misuse of prescription opioid pain relievers)	737 (2.2)	6,241,696 (2.3)
Stimulant or psychoactive drug use (non-opioid)	1,073 (3.3)	6,185,580 (2.2)
Non-opioid prescription drug misuse	935 (2.8)	6,620,057 (2.4)
Marijuana use	2,496 (7.6)	16,406,616 (5.9)
Legal substance use (alcohol, tobacco, and/or inhalants)	4,214 (12.8)	32,779,966 (11.8)

Abbreviation: NSDUH: National Survey on Drug Use and Health.

1. The totals of unweighted and weighted respondents were used as denominators in all the percentage calculations.

2. Other race included American Indians or Alaska Natives, and Native Hawaiians.

3. Polydrug use was defined in this study as use or misuse of drugs from two or more of the five drug groups

4. Five drug groups were defined based on their popularity and legal status in a mutually exclusive hierarchical order.

More than half of respondents (56.4%) reported using at least one substance in the past month (but not within the past year), while an additional 24.3% reported past-year (but not past month) use. When weighted, this corresponds to 58.0% of the U.S. civilian, noninstitutionalized population aged 12 years or older who used at least one substance in the past month and an additional 21.4% reporting past year (but not past month) use (Table 1).

### Prevalence of polydrug use

Among the 32,893 survey respondents, 12.7% (n = 4,174) reported past-month polydrug use, and an additional 3.8% (n = 1,253) reported past-year (but not past month) polydrug use, totaling 16.5% who engaged in polydrug use (Table 1). When weighted, this represents 11.4% (n = 314,47,483) and 2.8% (n = 7,716,381) of the civilian, noninstitutionalized population aged 12 years or older in the US who engaged in past-month or past-year polydrug use respectively, totaling 14.2% nationally. Among the survey respondents, the prevalence of past-month and past-year opioid use was 0.9% and 2.2%, respectively, with national estimates of 0.9% (n = 2,397,454) and 2.3% (n = 6,241,696; Table 1).

### Polydrug use by categories

Among the 18,545 respondents who used drugs in the past month, legal substances such as alcohol and tobacco were the most used substances (69.3%) (Table 2). Marijuana was the second most common drug used without other higher-category drugs (23.8%), while opioid use was the least common (1.6%). Additionally, among respondents who reported past-month use of opioids, stimulant or psychoactive drugs, marijuana, or misuse of non-opioid prescription drugs, 89.2%, 91.8%, 87.0%, and 97.9%, respectively, reported engaging in polydrug use.

**Table 2:** Prevalence of single drug and polydrug use among the respondents who used at least one of five drug groups, 2020 NSDUH.

Five Drug Groups^1^	Prevalence^2^, n (%)	Single or Polydrug Use^3^	
**Single, n (%)**	**Poly, n (%)**
**Past-Month Drug Use (n = 18,545)**			
Opioid use (heroin and misuse of prescription opioid pain relievers)	288 (1.6)	31 (10.8)	257 (89.2)
Stimulant or psychoactive drug use (non-opioid)	413 (2.2)	34 (8.2)	379 (91.8)
Non-opioid prescription drug misuse	573 (3.1)	12 (2.1)	561 (97.9)
Marijuana use	4,421 (23.8)	579 (13.1)	3,842 (87.0)
Legal substance use (alcohol, tobacco, and/or inhalants)	12,850 (69.3)		
**Past-Year Drug Use (but not past month) (n = 7,987)**			
Opioid use (heroin and misuse of prescription opioid pain relievers)	737 (9.2)	383 (52.0)	354 (48.0)
Stimulant or psychoactive drug use (non-opioid)	1,073 (13.4)	580 (54.1)	493 (46.0)
Non-opioid prescription drug misuse	935 (11.7)	428 (45.8)	507 (54.2)
Marijuana use	2,496 (31.3)	1,733 (69.4)	763 (30.6)
Legal substance use (alcohol, tobacco, and/or inhalants)	2,746 (34.4)		

Abbreviation: NSDUH: National Survey on Drug Use and Health

1. Five drug groups were defined based on their popularity and legal status in a mutually exclusive hierarchical order.

2. The prevalence of each of the five drug groups among respondents who engaged in drug use past-year or past-month.

3. Row percentages were presented among respondents who engaged in each of the five drug groups.

Among the 7,987 survey respondents who used drugs in the past year (but not past month), legal substances remained the most used (34.4%) (Table 2), followed by marijuana use (31.3%), hile opioid use remained the least common (9.2%). Additionally, among respondents who reported past-year use of opioids, stimulants or psychoactive drugs, marijuana, or misuse of non-opioid prescription drugs, 48.0%, 46.0%, 30.6%, and 54.2%, respectively, reported engaging in polydrug use.

### Polydrug use involving opioids

Of the 4,174 respondents who reported past-month polydrug use, 6.2% used opioids. Of the 1,253 respondents who reported past-year (but not past month) polydrug use, 28.3% used opioids (Table 3); when weighted, it represents national estimates of 7.0% and 32.5% opioid use among individuals who reported past-month and past-year polydrug use. Among those with past-month polydrug use, the weighted drug use percentages were 1.6% for opioids and stimulants or psychoactive drugs, 1.1% for opioids and misused non-opioid prescription drugs, and 4.3% for opioids with marijuana and/or legal drugs. Additionally, weighted percentages of respondents’ past-month polydrug use included 11.3% stimulant or psychoactive drug use without opioids, 6.1% non-opioid prescription drug misuse with marijuana and/or legal substance use, and 75.7% marijuana and legal drugs only (Table 3).

**Table 3:** Polydrug use patterns among respondents who engaged in polydrug use during past-month or past-year, 2020 NSDUH.

		Frequency	
		Unweighted, n (%)	Weighted, n (%)
**Past-Month Polydrug Use Pattern** ^**1**^			
Total Respondents Who Engaged in Past-month Polydrug Use		4,174	3,1447,484
A. Opioid use (heroin and misuse of opioid prescription pain relievers)	Overall	257 (6.2)	2,207,488 (7.0)
	+ Stimulant or psychoactive drug use	72 (1.7)	515,943 (1.6)
	+ non-opioid prescription drug misuse	50 (1.2)	332,531 (1.1)
	+ marijuana and/or legal substance use	135 (3.2)	1,359,014 (4.3)
B. Stimulant or psychoactive drug use (non-opioid)	Overall	489 (11.7)	3,538,177 (11.3)
	+ non-opioid prescription drug misuse	81 (1.9)	536,695 (1.7)
	+ marijuana and/or legal substance use	408 (9.8)	3,001,482 (9.5)
C. Non-opioid prescription drug misuse	+ marijuana and/or legal substance use	215 (5.2)	1,906,053 (6.1)
D. Marijuana use	+ legal substance use	3,213 (77.0)	23,795,766 (75.7)
**Past-Year Polydrug Use (but not past month) Pattern** ^**1**^			
Total Respondents Who Engaged in Past-year Polydrug Use		1,253	7,716,381
A. Opioid use (heroin and misuse of opioid prescription pain relievers)	Overall	354 (28.3)	2,511,095 (32.5)
	+ Stimulant or psychoactive drug use	125 (10.0)	800,620 (10.4)
	+ non-opioid prescription drug misuse	113 (9.0)	708,386 (9.2)
	+ marijuana and/or legal substance use	116 (9.3)	1,002,089 (13.0)
B. Stimulant or psychoactive drug use (Non-opioid)	Overall	368 (29.4)	2,401,845 (31.1)
	+ non-opioid prescription drug misuse	171 (13.7)	962,559 (12.5)
	+ marijuana and/or legal substance use	197 (15.7)	1,439,286 (18.7)
C. Non-opioid prescription drug misuse	+ marijuana and/or legal substance uses	174 (13.9)	1,038,162 (13.5)
D. Marijuana use	+ legal substance use	357 (28.5)	1,765,280 (22.9)

Abbreviation: NSDUH: National Survey on Drug Use and Health

1. Polydrug use patterns were defined hierarchically and mutually exclusively, ensuring that each respondent was assigned to only one pattern based on the specific drug groups involved.

Among those with past-year polydrug use (but not past month), the weighted percentages included 10.4% who used opioids and stimulants or psychoactive drugs concurrently, 9.2% who used opioids and misused non-opioid prescription drugs, and 13.0% who used opioids with marijuana and/or legal drugs. Additionally, weighted percentages of respondents’ past-year polydrug use, included 31.1% stimulant or psychoactive drug use without opioid use, 12.5% stimulant or psychoactive drug use and misuse of non-opioid prescription drugs concurrently, 13.5% misuse of non-opioid prescription drugs without opioids or stimulants or psychoactive drugs with marijuana and/or legal drugs, and 22.9% use of marijuana and legal substances only.

#### Associations between the first drug used and polydrug use/polydrug use with opioid involvement.

After adjusting for demographics, respondents who initiated drug use with opioids had over five times the odds to engage in past-month polydrug use (aOR = 5.45; 95% CI = 2.61, 11.37) and more than 15 times the odds to engage in past-month polydrug use with opioid involvement (aOR = 15.56; 95% CI = 4.92, 49.18) compared to those whose first drug was legal substances (Table 4).

**Table 4:** Associations between respondents’ first drug used and polydrug use and polydrug use with opioid involvement, 2020 NSDUH.

	Past-Month Use		Past-Year Use (but not past month)	
	Polydrug use vs Single drug use	Polydrug use with vs. without opioid involvement	Polydrug use vs Single drug use	Polydrug use with vs. without opioid involvement
	OR (95%CIs)^1^	OR (95%CIs)^1^	OR (95%CIs)^1^	OR (95%CIs)^1^
**First Drug Used** ^**2**^				
Opioid use (heroin and misuse of opioid prescription pain relievers)	**5.45 (2.61-11.37)**	**15.56 (4.92-49.18)**	**5.58 (2.76-11.29)**	**54.66 (23.80-125.55)**
Stimulant or psychoactive drug use (non-opioid)	**6.95 (4.53-10.67)**	1.77 (0.60-5.20)	**4.21 (2.76-6.44)**	**4.39 (2.42-7.97)**
Non-opioid prescription drug misuse	**2.77 (1.40-5.49)**	1.52 (0.34-6.86)	**7.17 (3.81-13.47)**	**8.05 (3.49-18.53)**
Marijuana use	0.86 (0.61-1.21)	**0.16 (0.03-0.94)**	**1.83 (1.12-3.00)**	1.05 (0.39-2.84)
Legal substance use (alcohol, tobacco, and/or inhalants)	Ref.	Ref.	Ref.	Ref.
**Sex**				
Male	Ref.	Ref.	Ref.	Ref.
Female	**0.80 (0.70-0.92)**	1.50 (0.93-2.43)	0.91 (0.75-1.10)	0.79 (0.50-1.23)
**Age**				
12-17	Ref.	Ref.	Ref.	Ref.
18-25	1.15 (0.86-1.54)	1.04 (0.47-2.31)	1.12 (0.80-1.57)	1.54 (0.58-4.08)
26-34	0.78 (0.58-1.05)	1.75 (0.76-4.04)	0.81 (0.59-1.12)	1.85 (0.72-4.78)
35-49	**0.51 (0.39-0.66)**	**2.53 (1.17-5.49)**	**0.71 (0.52-0.96)**	**4.75 (1.80-12.51)**
50 or older	**0.26 (0.19-0.35)**	**3.22 (1.45-7.12)**	**0.26 (0.16-0.43)**	1.56 (0.51-4.76)
**Race/Ethnicity**				
White Non-Hispanic	Ref.	Ref.	Ref.	Ref.
Black Non-Hispanic	**1.21 (1.00-1.46)**	1.05 (0.50-2.23)	**0.55 (0.34-0.89)**	1.45 (0.57-3.70)
Other race Non-Hispanic^3^	**0.74 (0.57-0.95)**	1.04 (0.25-4.29)	**0.56 (0.33-0.94)**	1.07 (0.35-3.29)
Multiple-race Non-Hispanic	**1.70 (1.10-2.65)**	0.70 (0.22-2.26)	0.83 (0.57-1.21)	0.43 (0.16-1.17)
Hispanic	**0.71 (0.57-0.88)**	1.10 (0.61-1.99)	**0.66 (0.49-0.89)**	0.62 (0.36-1.07)

Abbreviations: NSDUH: National Survey on Drug Use and Health; OR: Odds Ratios; 95%CI: 95% confidence intervals. Bold indicates statistical significance.

1. Polydrug use vs. single drug use, or Polydrug use with opioid involvement vs. without opioid involvement was modeled; Weighted data were used in model test.

2. First time use of a drug among the five drug groups.

3. Other race included American Indian or Alaska Native, and Native Hawaiian.

The odds of past-month polydrug use were: 6.95 times higher (95% CI: 4.53, 10.67) for people who initiated with stimulants or psychoactive drugs and 2.77 times higher (95% CI: 1.40, 5.49) for those who initiated with a misused non-opioid prescription drug, compared to those whose first drug was legal substances. However, the odds of past-month polydrug use involving opioids was relatively lower and not statistically significant for those who initiated with stimulants or psychoactive drugs or non-opioid prescription drug misuse. For individuals who initiated drug use with marijuana, there was a protective effect (aOR = 0.16, 95% CI: 0.03, 0.94).

Similar patterns were observed for past-year (but not past month) polydrug use and polydrug use with opioid involvement (Table 4). Compared to those whose first drug was legal substances, respondents who initiated drug use with opioids had over five times the odds of engaging in past-year polydrug use (aOR = 5.58; 95% CI = 2.76, 11.29) and more than 50 times the odds of engaging in past-year polydrug use with opioid involvement (aOR = 54.66; 95% CI = 23.80, 125.55), after adjusting for demographics. The odds of past-year polydrug use was: 4.21 times higher (95% CI = 2.76, 6.44) for those who started with stimulants or psychoactive drugs, 7.17 times higher (95% CI = 3.81, 13.47) for those who started with a misused non-opioid prescription drug, and 1.83 times higher (95% CI = 1.12, 3.00) for those who started with marijuana, compared to those whose first drug was legal substances. Similarly, the odds of past-year polydrug use involving opioids was: 4.39 times higher (95% CI = 2.42, 7.97) for those who started with stimulants or psychoactive drugs, and 8.05 times higher (95% CI = 3.49, 18.53) for those who started with a misused non-opioid prescription drug, compared to those whose first drug was legal substances.

Those identifying as female and as Hispanic had lower odds of both past-month and past-year polydrug use compared to their respective counterparts (Table 4). Those identifying as Non-Hispanic Black had significantly higher odds of past-month polydrug use but lower odds of past-year polydrug use compared to those identifying as Non-Hispanic White. Additionally, individuals aged 35–49 years had lower odds of past-month or past-year polydrug use but higher odds of past-month or past-year polydrug use involving opioids compared to those aged 12–17 years. Similar patterns were observed in individuals aged 50 or older as compared to those aged 12–17 years, although odds of past year polydrug use involving opioids was not statistically significant.

## Discussion

Understanding the prevalence and patterns of polydrug use, particularly those involving opioids, is crucial for planning interventions and treatment programs and addressing healthcare system demands. Built on limited previous studies examining concurrent polydrug use in the general population [14,15], this study used a large nationally representative sample to explore the prevalence of polydrug use involving opioids and the influence of the type of the first drug used on the likelihood of subsequent polydrug use and polydrug use involving opioids. Findings provide critical insights into patterns of polydrug drug use, including respondents’ timeframe of use (ongoing vs. past experimentation) and the involvement of opioids, with implications for public health strategies and future research aimed at addressing opioid use in the U.S. population.

Our results indicate that while the overall prevalence of polydrug use among individuals aged 12 and older was relatively low (11.4% for past-month use and an additional 2.8% for past-year use), polydrug use was common among respondents who used drugs. In our study, over four out of five people who used opioids, stimulants, psychoactive drugs, marijuana, or non-opioid prescription drugs in the past month—and nearly half of those who used them in the past year—reported using multiple drugs. A 2024 study found nearly 93% of fentanyl-positive specimens contained additional drugs [6]. The observed high prevalence of past-month polydrug use in our study indicates widespread ongoing, and higher-intensity short-term use, likely influenced by social contexts (e.g., peer settings or substance availability) and individuals’ efforts to enhance or modulate drug effects [29,39–41]. The near-universal polydrug use among respondents who misused prescription drugs (97.9%) observed in our study is particularly concerning, suggesting greater access to multiple substances or a higher-risk profile. Given that nearly half of 2019 drug overdose deaths involved multiple drugs according to Centers for Disease Control and Prevention (CDC) data [1], our findings highlight the urgent need for comprehensive prevention strategies to prevent drug use and reduce harms in those already using substances [6,21,24], ultimately protecting public health and minimizing the severe consequences of polydrug use.

Our results indicate that 1.6% of respondents who used at least one of five drug groups used opioids in the past month and an additional 9.2% of respondents used opioids in the past year (but not past month). Among people who used opioids in the past month and past year, 89.2% and 48.0%, respectively, reported using multiple substances. These findings align with data from the NSDUH from 2017–2019, which reported high rates of polydrug use among those who misuse opioids [42]. The involvement of opioids in stimulant-related deaths has increased, with nearly 73% of cocaine-involved overdose deaths also involving an opioid in 2017 [43]. Synthetic opioids are driving increases in cocaine-involved overdose deaths [44], with about one-third of psychostimulant-involved deaths also involving synthetic opioids in 2019 [45]. Our results show similar proportions of polydrug use among those who use opioids and those using other stimulants or psychoactive drugs, misusing prescription drugs, or using marijuana. Previous studies suggest that there is higher polydrug use when stimulants or psychoactive drugs are involved compared to when marijuana and legal substances are used [46,47]. Additional studies are needed to uncover patterns of polydrug use when engaging in various substances.

Individuals whose first drug was an opioid had more than five times the likelihood of engaging in both past-month and past-year polydrug use compared to those who started with a legal substance. This result suggests that early opioid exposure may strongly predict broader substance use patterns. The progression from one substance to another, often referred to as the gateway effect, may stem from neurobiological changes, genetic predisposition, environmental and behavioral factors, and the additive nature of substances [48–50]. Exposure to one substance may sensitize an individual to other substances [51], leading to further substance use [52]. Furthermore, our findings show that individuals who started with opioids had a 15-fold likelihood of engaging in past-month polydrug use involving opioids and 50-fold likelihood of engaging in past-year polydrug use involving opioids, suggesting that early opioid use is a strong predictor of polydrug use involving opioids. In contrast, respondents whose first drug was not an opioid had a much lower likelihood of later opioid use. These findings underscore the influential role of opioids in shaping long-term substance use trajectories. Early opioid exposure could increase the risk of polydrug use and reinforce opioid dependence, emphasizing the urgent need for preventive interventions to deter initial opioid use. Additionally, targeted efforts are essential to support individuals whose first drug is an opioid, helping prevent further opioid-related polydrug use and reducing the associated risks of overdose and death.

Our findings reveal distinct patterns in opioid-involved polydrug use based on both the type and timing of drug initiation. Individuals who began with stimulants, psychoactive substances, or misused prescription drugs were more likely to engage in past-year opioid-involved polydrug use compared with those who initiated with legal substances. In contrast, past-month opioid-involved polydrug use was lower and not statistically significant for these groups. Notably, individuals who initiated marijuana use showed a reduced likelihood of opioid-involved polydrug use in the past month. Several factors may explain these differences. Early initiation with stimulants, psychoactive substances, or prescription drug misuse is often associated with higher-risk trajectories characterized by experimentation, sensation seeking, or pharmacological motivations that increase vulnerability to opioid co-use [11,44]. These substances are also commonly combined with opioids to enhance euphoria, alleviate withdrawal, or modulate sedation [53]. In contrast, individuals who initiate with marijuana may represent a distinct subgroup with different social contexts and motivations, typically involving recreational or normative use within peer settings, resulting in a lower likelihood of transitioning to opioid misuse [54,55]. This apparent protective association may also reflect variations in drug availability, perceived risk, legalization status, and the characteristics of individuals who initiate drug use with marijuana compared to those who begin with other illicit or prescription drugs.

Consistent with prior national survey and epidemiologic findings, we also found that individuals identifying as Female and Hispanic had lower odds of both past-month and past-year polydrug use compared to their respective counterparts. Individuals identifying as female generally report lower rates and intensity of substance use due to sociocultural and biological factors, including stronger social deterrents, greater perceived stigma, heightened physiological sensitivity, and higher treatment engagement [56,57]. Similarly, Hispanic populations tend to show lower prevalence of substance and polydrug use compared with non-Hispanic White and Black populations—a pattern often described as the “Hispanic paradox” [58]. Protective factors such as family cohesion, religiosity, and community support, as well as reduced exposure to high-risk social environments, may contribute to this pattern [59]. Together, these mechanisms may help explain the lower likelihood of opioid-involved and broader polydrug use among these groups.

Research on polydrug use is challenging due to varying definitions, types of drugs, and populations studied [33]. While some define polydrug use as using multiple substances simultaneously or in close succession [60,61], others focus on specific drug combinations [62,63]. For example, using cigarettes and alcohol differs significantly from combining cocaine and marijuana, leading to distinct behaviors and consequences. Most studies define polydrug use based on their focus, whether general substance use or psychological effects. Our epidemiological study defined polydrug use as the use of two or more of five hierarchically and mutually exclusive drug groups. Our findings enhance the understanding of polydrug use prevalence, patterns, opioid involvement, and the first drug used.

### Limitations

As a secondary data analysis, our study was limited to the variables available in the dataset. First, past-month use of opioids, particularly prescription opioid misuse, was not collected. To address this, we used a reference method as a proxy for monthly use. Second, our operational definition of polydrug use may underestimate its prevalence for two reasons**:** 1) drug use was self-reported and therefore subject to potential social desirability bias**,** and 2) the use of multiple drugs within the same drug group was not classified as polydrug use; only the use of drugs across different groups was counted. This approach may obscure some within-group variation, such as concurrent use of methamphetamine and hallucinogens or of stimulants and tranquilizers. Additionally, our polydrug use patterns do not account for the pharmacological and psychological effects of the substances. Future research should incorporate these factors for a more comprehensive understanding of polydrug use patterns. Third, our findings are based solely on 2020 data and may not reflect trends in other years. NSDUH data do not include individuals who experience homelessness, active-duty military, or persons residing in institutions, such as those who are incarcerated; thus, polydrug use estimates in this study might not be generalizable to the total U.S. population. Fourth, estimates for fentanyl—particularly illicitly manufactured fentanyl—are relatively new to the NSDUH, with 2022 marking the first year national estimates were available [64]. Because our study used 2020 data, such information was unavailable, preventing us from examining specific patterns of fentanyl and methamphetamine co-use. Lastly, our study could not capture key details of respondents’ polydrug use, such as whether substances were used concurrently or sequentially, the quantities consumed, or the order of use.

## Conclusions

This study highlights the significant intersection between opioid use and polydrug use, emphasizing the heightened risk among individuals who initiate drug use with opioids. While overall opioid use remains low in the general population, its prevalence is alarmingly high among respondents engaging in polydrug use. Those identifying as male and those whose first drug use was an opioid are at increased risk of polydrug use, suggesting the need for targeted prevention efforts. Future research should explore factors driving polydrug use and the long-term impact of early initiation of opioid use. Public health efforts should focus on preventing or delaying opioid use onset and providing targeted interventions for high-risk groups. Tailored education, harm reduction strategies, and improved access to treatment are crucial for reducing opioid-involved polydrug use and its associated harms.
